# Direct Electrochemistry and Electrocatalysis of Horseradish Peroxidase Immobilized in a DNA/Chitosan-Fe_3_O_4_ Magnetic Nanoparticle Bio-Complex Film

**DOI:** 10.3390/ma7021069

**Published:** 2014-02-11

**Authors:** Tingting Gu, Jianli Wang, Hongqi Xia, Si Wang, Xiaoting Yu

**Affiliations:** School of Chemical Engineering, University of Science and Technology Liaoning, 185 Qianshan Road, Anshan 114051, Liaoning, China; E-Mails: ustlwangjianli@gmail.com (J.W.); ustlxiahongqi@gmail.com (H.X.); ustlwangsi@gmail.com (S.W.); strongerxt@gmail.com (X.Y.)

**Keywords:** horseradish peroxidase, direct electron transfer, DNA/chitosan polyion complex film, Fe_3_O_4_ magnetic nanoparticles, H_2_O_2_ biosensors

## Abstract

A DNA/chitosan-Fe_3_O_4_ magnetic nanoparticle bio-complex film was constructed for the immobilization of horseradish peroxidase (HRP) on a glassy carbon electrode. HRP was simply mixed with DNA, chitosan and Fe_3_O_4_ nanoparticles, and then applied to the electrode surface to form an enzyme-incorporated polyion complex film. Scanning electron microscopy (SEM) was used to study the surface features of DNA/chitosan/Fe_3_O_4_/HRP layer. The results of electrochemical impedance spectroscopy (EIS) show that Fe_3_O_4_ and enzyme were successfully immobilized on the electrode surface by the DNA/chitosan bio-polyion complex membrane. Direct electron transfer (DET) and bioelectrocatalysis of HRP in the DNA/chitosan/Fe_3_O_4_ film were investigated by cyclic voltammetry (CV) and constant potential amperometry. The HRP-immobilized electrode was found to undergo DET and exhibited a fast electron transfer rate constant of 3.7 s^−1^. The CV results showed that the modified electrode gave rise to well-defined peaks in phosphate buffer, corresponding to the electrochemical redox reaction between HRP(Fe^(III)^) and HRP(Fe^(II)^). The obtained electrode also displayed an electrocatalytic reduction behavior towards H_2_O_2_. The resulting DNA/chitosan/Fe_3_O_4_/HRP/glassy carbon electrode (GCE) shows a high sensitivity (20.8 A·cm^−2^·M^−1^) toward H_2_O_2_. A linear response to H_2_O_2_ measurement was obtained over the range from 2 μM to 100 μM (*R*^2^ = 0.99) and an amperometric detection limit of 1 μM (S/N = 3). The apparent Michaelis-Menten constant of HRP immobilized on the electrode was 0.28 mM. Furthermore, the electrode exhibits both good operational stability and storage stability.

## 1. Introduction

The direct electron transfer (DET) between electrodes and redox proteins, particularly enzymes, has stimulated increasing interest because of its significance in both theoretical and practical applications in electrochemistry, such as fabricating biosensors, enzymatic bioreactors, and biomedical devices [1–3]. Nevertheless, DET between the enzyme and a conventional electrode is usually prohibited because enzymatic redox centers are deeply embedded in the structure of the enzyme. Therefore, new materials, such as carbon nanotube [4–6], quantum dot [7], nanoparticles [8], graphene [4], hybrid organic-inorganic film [9], room temperature ionic liquid matrix [10] and mesoporous matrix [11] are required to establish an electrical connection between these redox centers and the electrode for fabricating a third-generation enzyme biosensor or a mediatorless enzymatic biofuel cell.

In recent years, there has been an increasing trend in the design and development of magnetic nanoparticles for bioanalytical applications [12,13]. Magnetic nanoparticles have been considered as an interesting material for the immobilization of desired biomolecules because of some superior properties: electro-conductivity, bio-compatibility and ease of synthesis [14]. Enzyme-immobilized magnetic nanoparticles could potentially lead to unique properties such as large surface area, high bioactivity, and excellent stability [15,16]. However, pure iron oxide nanoparticles may not be very useful in biomedical and technological applications because they are very likely to aggregate, and they have limited functional groups for selective binding [17]. As a result, a number of biomaterial-functionalized magnetic nanoparticles have been used in bioelectronic applications [12–17], such as poly(ethyleneimine) layer-functionalized magnetic nanoparticles [18], Au-polydopamine-Fe_3_O_4_ magnetic nanoparticles [19], and magnetic core-shell Fe_3_O_4_@Al_2_O_3_ nanoparticles [20], which were used for capture of heme proteins for direct electrochemistry. Unfortunately, these methods may involve complicated electrode modification procedure or costly modification regents.

DNA/polycation based biocompatible films are formed by natural DNA molecules (negatively charged polyanions) and natural polycations molecules based on the electrostatic force of attraction. As novel electrochemical recognition layers, DNA/polycation based biocompatible films possess a number of unique properties [21–23]: (1) biocompatible microenvironment around the enzyme; (2) a host matrix of electrochemically active species (e.g., redox active intercalators) and metal ions which specifically binds to double-stranded DNA; (3) unique electron transfer property improving electron transfer characteristics between redox active species and the electrode surface; and (4) simplicity in procedure. In our previous reports, DNA/poly(allylamine) films have been successfully used as the support matrixes for co-immobilization of electron mediator-methylene blue and HRP [22–24], and immobilization of electrocatalytic element-copper ion [23,25] to fabricate novel amperomeric biosensors. More recently, glucose oxidase (GOD) was effectively immobilized on a DNA/chitsoan films modified glassy carbon electrode (GCE), and the direct electrochemistry of GOD and biosensing for glucose were performed successfully [26].

The DET of immobilized heme proteins such as horseradish peroxidase (HRP) [3], cytochrome C [27], and hemoglobin [28] is based on the Fe^(III)^/Fe^(II)^ conversion in the active heme center of the proteins. Among them, HRP has been widely used for the fabrication of amperometric biosensors based on its direct electrochemistry to detect H_2_O_2_ due to its high purity, sensitivity, low cost and availability [29]. There are many ways to achieve the DET of immobilized HRP, for instance, using sol-gel matrices [30], biopolymers [31], and nanomaterials [32].

In this paper, a new type of DNA/chitosan/Fe_3_O_4_ magnetic nanoparticle bio-complex film was constructed for the immobilization of HRP. DNA/chitsoan film and Fe_3_O_4_ nanoparticles with the excellent biocompatibility and good conductivity were used to maintain the native structure of HRP and to facilitate the direct electrochemistry of HRP in the biofilm. Although a Fe_3_O_4_/chitosan/HRP-modified glassy carbon electrode has been reported for amperometric detection of H_2_O_2_ [33], methylene blue in solution was needed as an electron transfer mediator to transfer the electron between the HRP and electrode in the system. Here, the direct electron transfer and electrocatalysis of HRP based on the DNA/chitosan/Fe_3_O_4_ film was studied.

## 2. Results and Discussion

### 2.1. Morphologies of DNA/Chitosan/Fe_3_O_4_/HRP Film Surface

**Figure 1.**
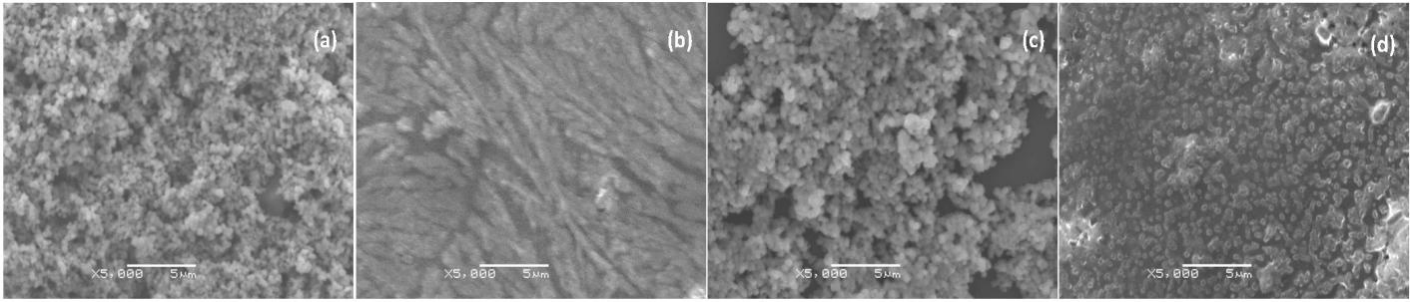
Scanning electron micrographs of (**a**) Fe_3_O_4_-; (**b**) DNA/chitosan-; (**c**) DNA/chitosan/Fe_3_O_4_-; and (**d**) DNA/chitosan/Fe_3_O_4_/horseradish peroxidase (HRP)-modified glassy carbon electrodes.

The surface morphologies of Fe_3_O_4_, DNA/chitosan, DNA/chitosan/Fe_3_O_4_ and DNA/chitosan/Fe_3_O_4_/HRP were examined by scanning electron microscopy (SEM). The micrograph of immobilized Fe_3_O_4_ shows aggregated particles with diameter of approximately 0.2 μm (Figure 1a). The micrograph of a DNA/chitosan film appears to be a smooth surface (Figure 1b), and that of a DNA/chitosan/Fe_3_O_4_ film shows uniformly distributed particles of approximately 0.4 μm diameter (Figure 1c). In contrast, following HRP immobilization, DNA/chitosan/Fe_3_O_4_/HRP film (Figure 1d) shows a rough surface with randomly distributed small spots, indicating that HRP was entrapped in the DNA/chitosan/Fe_3_O_4_ films. This is further supported by an increased electron transfer resistance of DNA/chitosan/Fe_3_O_4_/HRP-modified electrode reported in Section 2.2.

**(a)**

**(b)**

**(c)**

**(d)**

### 2.2. Electrochemical Impedance Spectroscopy (EIS) of DNA/Chitosan/Fe_3_O_4_/HRP Film

**Figure 2.**
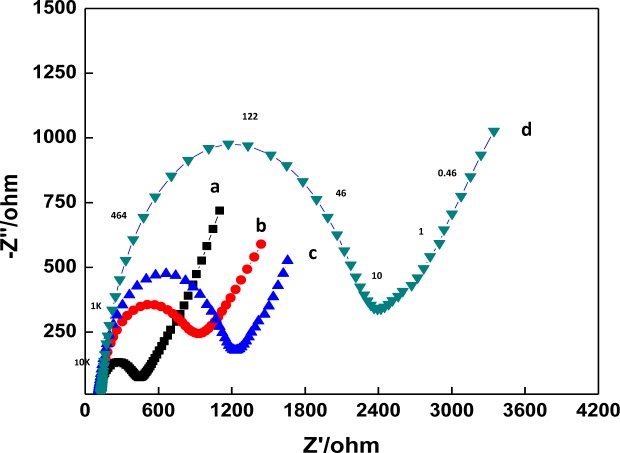
Electrochemical Impedance Spectroscopy (EIS) characterization of different modified electrodes: (**a**) bare glassy carbon electrode (GCE); (**b**) DNA/chitosan/Fe_3_O_4_/GCE; (**c**) DNA/chitosan/GCE; and (**d**) DNA/chitosan/Fe_3_O_4_/HRP/GCE in 0.1 M KCl solution containing 5 mM [Fe(CN)_6_]^3−/4−^. Applied potential was set at 170 mV *vs.* Ag/AgCl (formal potential of [Fe(CN)_6_]^3−/4−^) with the frequency range from 0.01 to 100,000 Hz with the amplitude of 8 mV. Representative frequencies are as indicated in curve (**d**).

EIS can provide useful information on the impedance changes of the electrode surface during the fabrication process. In EIS, the diameter of a semicircle in the high frequency region corresponds to the electron transfer resistance, *R*_et_. This resistance controls the electron transfer kinetics of redox probe at the electrode interface. Figure 2 displays the Nyquist plots of the EIS of GCE (a), a DNA/chitosan/GCE (b), a DNA/chitosan/Fe_3_O_4_/GCE (c) and a DNA/chitosan/Fe_3_O_4_/HRP/GCE (d) in 0.1 M KCl containing 5.0 mM K_3_Fe(CN)_6_/K_4_Fe(CN)_6_. The bare GCE reveals a very small semicircle domain (curve a, *R*_et_ = 446 Ω). After DNA/chitosan film was immobilized on the GCE, an increase of *R*_et_ to 1237 Ω was observed (curve b), which indicated that the DNA/chitostan polyion complex film hindered the electron transfer of K_3_Fe(CN)_6_/K_4_Fe(CN)_6_. For DNA/chitosan/Fe_3_O_4_/GCE, the value of *R*_et_ was found to be 940 Ω, implying that the incorporation of Fe_3_O_4_ facilitated electron transfer (curve c). There was further immobilization of enzymes: the *R*_et_ of DNA/chitosan/Fe_3_O_4_/HRP/GCE increased to 2392 Ω, which was caused by the hindrance of the macromolecular structure of HRP to the electron transfer. The above results clearly confirm that HRP was immobilized successfully onto the electrode.

### 2.3. Direct Electrochemistry of HRP at DNA/Chitosan/Fe_3_O_4_ Film

**Figure 3.**
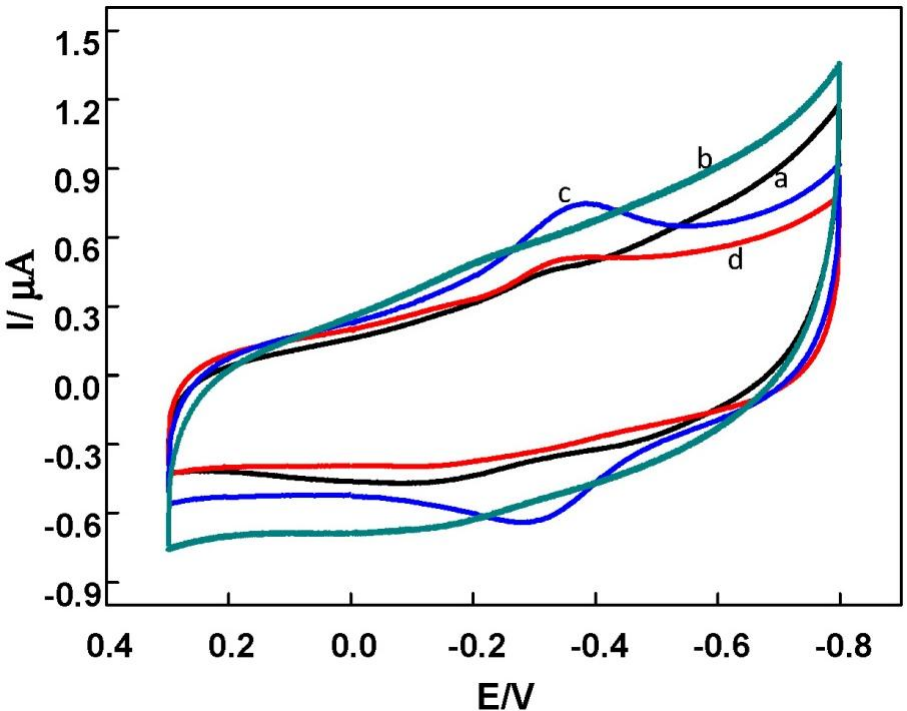
Cyclic voltammograms (CVs) of the different modified GC electrodes in N_2_-saturated phosphate buffer solution (pH 7.0) at scan rate of 150 mV/s. (**a**) DNA/chitosan/GCE; (**b**) DNA/chitosan/Fe_3_O_4_/GCE; (**c**) DNA/chitosan/Fe_3_O_4_/HRP/GCE; (**d**) DNA/chitosan/HRP/GCE.

Figure 3 shows the cyclic voltammograms (CVs) of DNA/chitosan/GCE, DNA/chitosan/Fe_3_O_4_/GCE, DNA/chitosan/HRP/GCE and DNA/chitosan/Fe_3_O_4_/HRP/GCE in N_2_-saturated phosphate buffer. DNA/chitosan/GCE (curve a) and DNA/chitosan/Fe_3_O_4_/GCE (curve b) did not show any redox wave in the potential range studied. In contrast, a pair of well-defined quasi-reversible redox peaks was obtained at the −0.35 V and −0.30 V by DNA/chitosan/Fe_3_O_4_/HRP/GCE (Figure 3c). The formal potential (E^0^′) was estimated to be ~ −0.32 V (*versus* Ag/ACl in saturated KCl) by the average of the cathodic and anodic peak potentials. Therefore, it can be concluded that the redox waves should be ascribed only to HRP, which is characteristic of quasi-reversible DET process of HRP[Fe^(III)^] and HRP[Fe^(II)^] in the HRP previously reported in various films [30–32]. Thus, DET of HRP in the DNA/chitosan/Fe_3_O_4_ film has been achieved successfully. Similar to the case of DNA/chitosan/Fe_3_O_4_/HRP/GCE (curve c), the DNA/chitosan/HRP modified electrode (curve d) showed a pair of quasi-reversible redox peaks, but the oxidation and reduction peak currents were 87% and 53%, respectively, smaller than the corresponding peak currents obtained at DNA/chitosan/Fe_3_O_4_/GCE. This result clearly indicated that the electron transfer of HRP on the DNA/chitosan electrode was amplified by incorporation of Fe_3_O_4_ magnetic nanoparticles.

**Figure 4.**
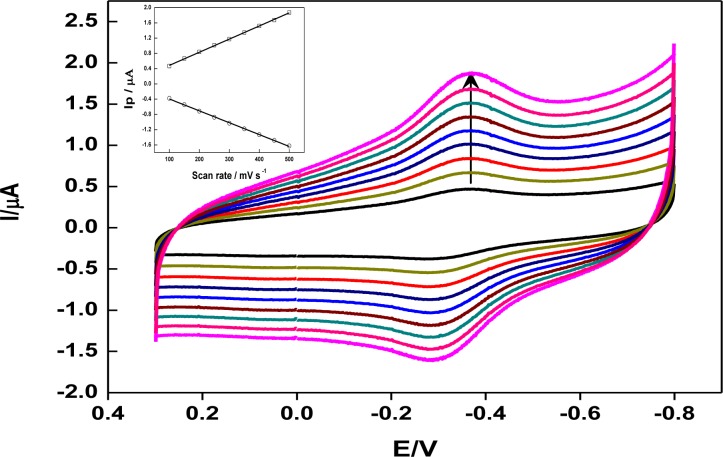
Cyclic voltammograms of DNA/chitosan/Fe_3_O_4_/HRP/GCE in N_2_-saturated phosphate buffer solution (pH 7.0) at different scan rates and (inset) plots of peak currents *vs.* scan rates. The scan rates (from inner to outer) are 100, 150, 200, 250, 300, 350, 400, 450 and 500 mV·s^−1^, respectively.

The cyclic voltammograms of DNA/chitosan/Fe_3_O_4_/HRP/GCE at various scan rates were investigated (Figure 4). The linear relationship between peak current and scan rate indicated that the redox process of HRP in the film was a surface-confined process. The electron transfer rate constant (*k*_s_) has been estimated from the peak potential separation value using the model of Laviron [34]. Taking a charge transfer coefficient α of 0.5, the electron transfer rate constant of HRP at the DNA/chitosan/Fe_3_O_4_/HRP/GCE was 3.7 s^−1^. The electron transfer rate is higher than the values reported for HRP immobilized in a polystyrene and multiwalled carbon nanotube composite film (1.15 s^−1^) [32], a colloidal gold modified screen-printed electrode (0.75 s^−1^) [35], and a dipalmitoyl phosphatidic acid film (1.13 s^−1^) [36]. Since DNA/chitsoan film and Fe_3_O_4_ nanoparticles with the excellent biocompatibility and good conductivity appeared to be capable of maintaining the native structure of HRP, it suggested that DNA/chitosan/Fe_3_O_4_ film was an excellent promoter for the direct electron transfer between HRP and GCE.

Based on the equation, *Q =* *nFAΓ*, where *n* denotes the charge of the redox reaction, *Q* denotes the quantity of electricity, *F* denotes the Faraday constant and *A* denotes the area of the electrode surface (in this case, *A* = 0.02 cm^2^), the surface coverage (*Γ*_HRP_) of HRP was estimated to be 3.5 × 10^−10^ mol·cm^−2^ at the DNA/chitosan/Fe_3_O_4_/HRP/GCE. The relative amount of electroactive HRP was 36.1% of the total amount of HRP deposited on the electrode surface. The results demonstrated that the composite film is efficiency for HRP immobilization.

**Figure 5.**
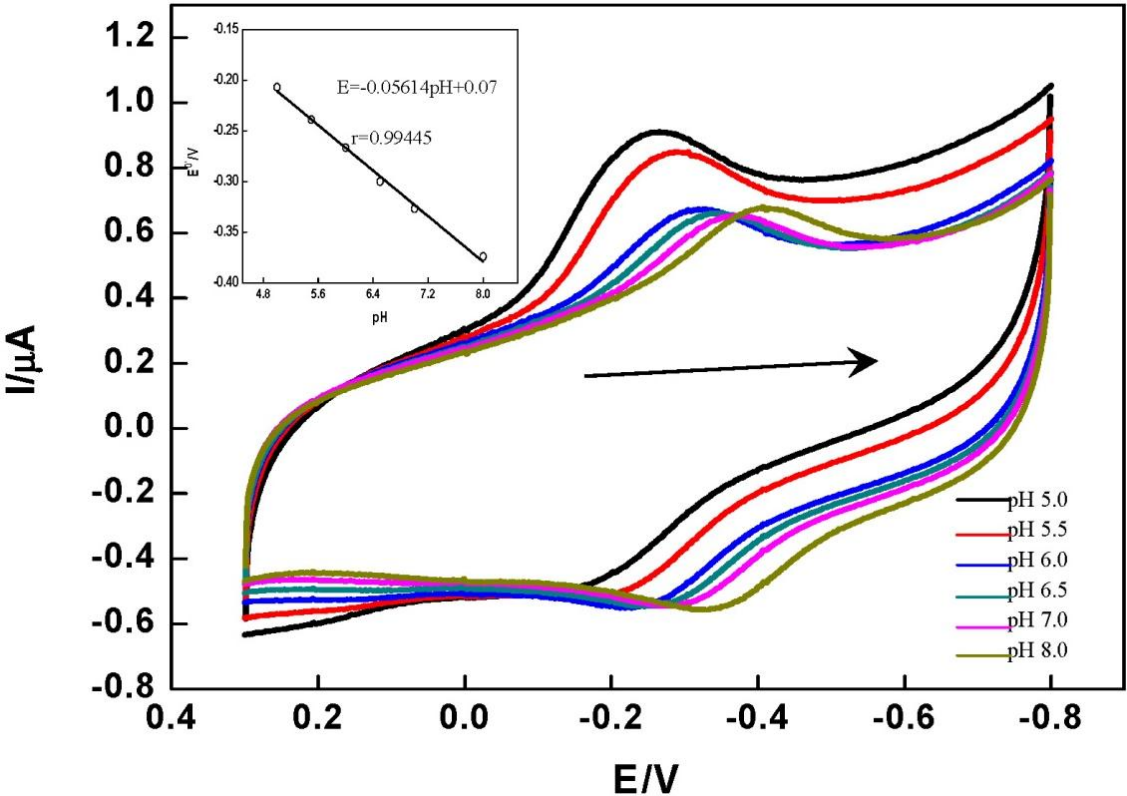
Cyclic voltammograms of DNA/chitosan/Fe_3_O_4_/HRP/GCE in different pH phosphate buffers (from left to right 5.0, 5.5, 6.0, 6.5, 7.0, 8.0). Scan rate: 150 mV·s^−1^. Inset: the plot of E^0^′ of HRP *vs.* pH of the solution.

The DET of HRP immobilized on the DNA/chitosan/Fe_3_O_4_/HRP/GCE showed a strong dependence on solution pH. Figure 5 shows the CVs of the DNA/chitosan/Fe_3_O_4_/HRP/GCE in phosphate buffer at different pH values. CVs with stable and well defined peaks were observed in the pH range 5.0–8.0, but increasing pH caused a negative shift of both cathodic and anodic peak potentials. This is attributed to the involvement of proton transfer in the HRP[Fe^(III)^]/HRP[Fe^(II)^] redox couple. The E^0^′ value of HRP varied linearly in the range of pH from 5.0 to 8.0, with a slope of 56.14 mV pH^−1^ (inset graph in Figure 5). This value is very close to the theoretical value for the transfer of one proton and one electron in a reversible reduction (58 mV pH^−1^ at 25 °C) [37].

### 2.4. Electrocatalysis of DNA/Chitosan/Fe_3_O_4_/HRP/GCE

**Figure 6.**
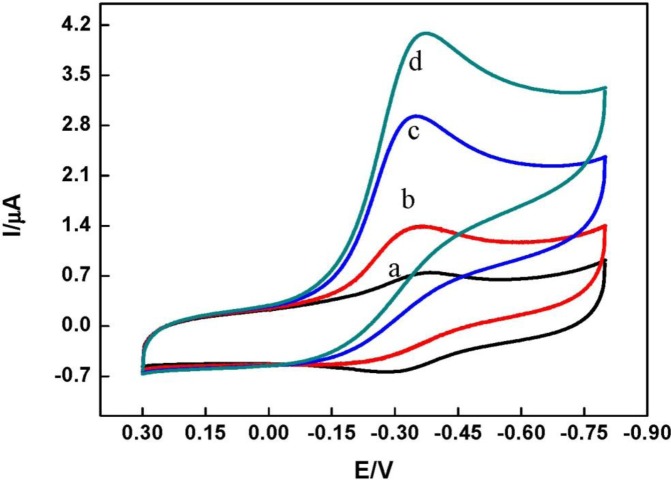
Cyclic voltammograms of DNA/chitosan/Fe_3_O_4_/HRP/GCE in N_2_-saturated phosphate buffer solution (pH 7.0) in the presence of various H_2_O_2_ concentrations. (**a**) 0 mM; (**b**) 1 mM; (**c**) 3 mM; (**d**) 4 mM.

By using hydrogen peroxide as a probe, the electrocatalytic properties of the HRP *in* DNA/chitosan/Fe_3_O_4_/GCE were studied. Figure 6 shows the bioelectrocatalytic activity of the HRP *in* DNA/chitosan/Fe_3_O_4_/GCE toward the reduction of H_2_O_2_ at a scan rate of 0.1 V·s^−1^. A pair of quasi-reversible CV peaks appeared in the absence of H_2_O_2_ (curve a). Upon the addition of H_2_O_2_ to the pH 7.0 phosphate buffer, the reduction peak current of the immobilized HRP increased dramatically and the oxidation peak current decreased concomitantly (curve b, c and d). The reduction peak current increases with increasing concentration of H_2_O_2_ (curve b, c and d), indicating that an electrocatalytic reduction of H_2_O_2_ took place at electrode and the HRP entrapped in the DNA/chitosan/Fe_3_O_4_/HRP film maintained its bio-electrocatalytic activity. Similar electrocatalytic behavior has been reported at Nafion/HRP/graphene/GCE [3] and HRP-incorporated in dipalmitoyl phosphatidic acid film-modified pyrolytic graphite electrode [36].

The electrocatalytic mechanism of HRP immobilized in the DNA/chitosan/Fe_3_O_4_/HRP film to H_2_O_2_ reduction can be represented as follows [38]:

$$\mathrm{HRP}[{\mathrm{Fe}}^{\left(\mathrm{III}\right)}]+H_{2}O_{2}\to \mathrm{Compound}\;I+H_{\text{2}}O$$ (1)

$$\mathrm{Compound}\;I+e^{-}+H^{+}\to \mathrm{Compound}\;\mathrm{II}$$ (2)

$$\mathrm{Compound}\;\mathrm{II}+e^{-}+H^{+}\to \mathrm{HRP}[{\mathrm{Fe}}^{\left(\mathrm{III}\right)}]+H_{2}O$$ (3)

here, HRP contains heme as an active site; in the resting state, the heme-iron oxidation state is HRP[Fe^(III)^]. The basic catalytic mechanism of HRP occurs through the rapid reaction with H_2_O_2_ to give a two-equivalent oxidized form, called Compound I as shown in Equation (1); the rapid reaction of compound I with the substrate then regenerates the HRP[Fe^(III)^] ground state form via an intermediate called Compound II, as shown in Equations (2) and (3) [39].

**Figure 7.**
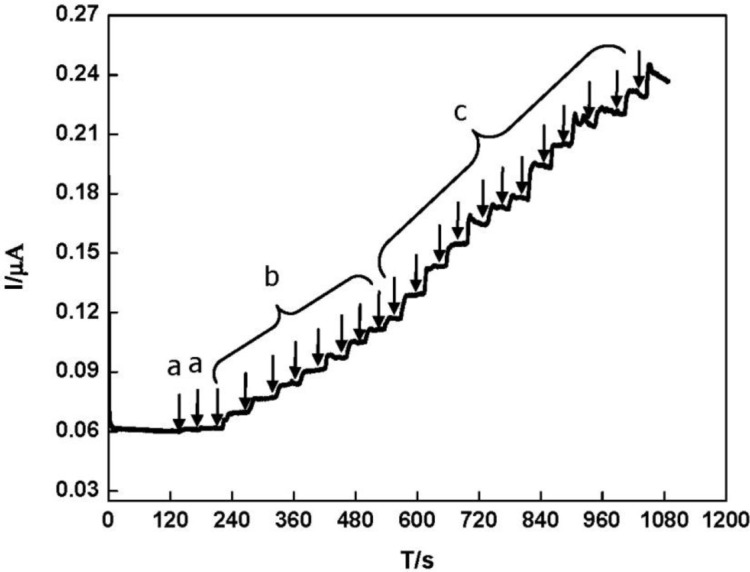
Typical current-time response curve of DNA/Fe_3_O_4_/chitosan/HRP/GCE for the successive addition of H_2_O_2_ in phosphate buffer solution (pH 7.0) at the constant electrode potential of −0.25 V. H_2_O_2_ concentration: (**a**) 1 μM; (**b**) 2 μM; (**c**) 5 μM.

The electrocatalytic property of the DNA/chitosan/Fe_3_O_4_/HRP/GCE was also studied by the constant potential amperometry. To reduce the background current, we selected −0.25 V as an applied potential. Figure 7 illustrates a typical current-time curve of the DNA/chitosan/Fe_3_O_4_/HRP/GCE at −0.25 V with successive addition of H_2_O_2_. When H_2_O_2_ was added to the stirred phosphate buffer, the reduction current increased rapidly and 95% of the steady-state current was obtained within 6 s. The H_2_O_2_ reduction current at the DNA/chitosan/Fe_3_O_4_/HRP/GCE changes linearly from 2 μM to 100 μM with a correlation coefficient of 0.99 (*n* = 3). Using the linear calibration plot, we have estimated a detection limit of 1 μM, based on S/N = 3.

**Figure 8.**
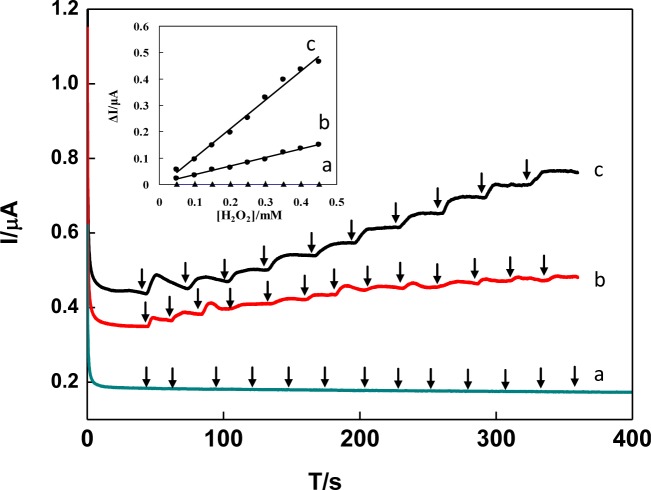
Typical current-time response curve for successive addition of 0.1 mM H_2_O_2_ obtained by DNA/chitosan/GCE (**a**), DNA/chitosan/HRP/GCE (**b**) and DNA/chitosan/Fe_3_O_4_/HRP/GCE (**c**) in 0.1M phosphate buffer (pH 7.0) at applied potential of −0.25 V. Inset: the plots of amperometric responses (Δ*I*) *vs.* H_2_O_2_ concentration.

Figure 8 shows the comparison of amperometric current-time curves for successive addition of 0.1 mM H_2_O_2_ obtained by DNA/chitosan/GCE (a), DNA/chitosan/HRP/GCE (b) and DNA/chitosan/Fe_3_O_4_/HRP/GCE (c) at applied potential of −0.25 V (*versus* Ag/AgCl). The observed catalytic responses of electrodes were in the following order: DNA/chitosan/Fe_3_O_4_/HRP/GCE > DNA/chitosan/HRP/GCE > DNA/chitosan/GCE. No response was observed on the DNA/chitosan/GCE without HRP (Figure 8a), indicating that direct catalyzed reduction of H_2_O_2_ was negligible. The amperometric responses of DNA/chitosan/HRP/GCE (Figure 8b) were observed for 0.1 mM H_2_O_2_, which was contributed from the direct electron transfer between HRP embedded in the DNA/chitosan film and GCE. When Fe_3_O_4_ was present in the DNA/chitosan/HRP/GCE, the sensitivity of H_2_O_2_ responses obviously increased from 8.86 A·cm^−2^·M^−1^ to 20.8 A·cm^−2^·M^−1^. These results indicated that Fe_3_O_4_ nanoparticles in the film play an important role to enhance the DET and electrocatalytic properties of HRP molecules.

**Figure 9.**
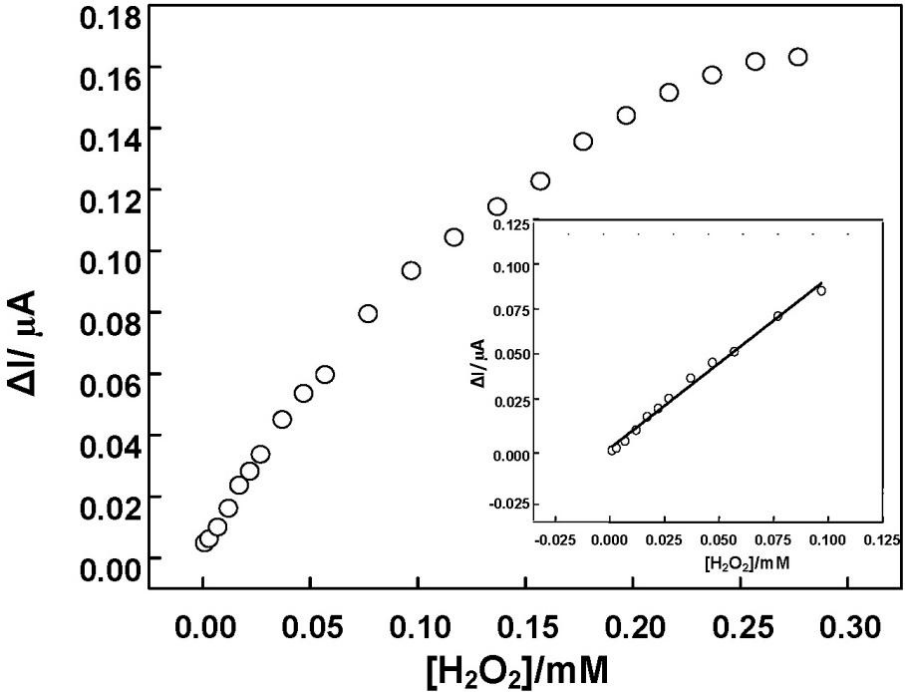
The calibration curve of amperometric response of DNA/chitosan/Fe_3_O_4_/HRP/GCE to H_2_O_2_ concentration at the constant electrode potential of −0.25 V. Measurement conditions were the same as in Figure 8.

Figure 9 shows the calibration curve of amperometric response of DNA/chitosan/Fe_3_O_4_/HRP/GCE *versus* H_2_O_2_ concentration. The sensitivity of the DNA/chitosan/Fe_3_O_4_/HRP/GCE was calculated to be 20.8 A·cm^−2^·M^−1^. The relationship between the catalytic current and the concentration of H_2_O_2_ shows a Michaelis-Menten kinetic mechanism. The apparent Michaelis-Menten constant (*K*_m_) could be calculated from the electrochemical Lineweaver-Burk equation: 1/*I*_ss_ = (*K*_m_/*I*_ss_) × (1/*C*) + 1/*I*_max_, where *I*_ss_, *I*_max_ and *C* represent the steady current, maximum current and H_2_O_2_ concentration, respectively. According to the intercept and slope of above regression equation, *K*_m_ was estimated to be 0.28 mM. The value is much smaller than 0.66 mM for HRP immobilized in a polystyrene and multiwalled carbon nanotube composite film [32], and 0.818 mM for HRP immobilized in γ-Al_2_O_3_ nanoparticles/chitosan film [40]. The small apparent Michaelis-Menten constant shows a high affinity to H_2_O_2_ and good bioactivity of DNA/chitosan/Fe_3_O_4_/HRP/GCE toward H_2_O_2_ reduction.

The long-term stability of electrode was investigated by examining its current response after storage in a refrigerator at 4 °C. The electrode exhibited no obvious decrease in current response in the first week and maintained about 95% of its initial value after three weeks. The relative standard deviation (R.S.D.) of the electrode response to 10 mM H_2_O_2_ for 5 successive measurements was 2.1%, indicating an acceptable electrode reproducibility. The selectivity of electrode was performed by comparing the amperometric response of 0.2 mM H_2_O_2_ before and after adding 2 mM of several known interfering species, respectively, in 0.1 M phosphate buffer (pH 7.0). The steady-state amperometric current ratio obtained in the presence to that in the absence of each of these interfering species is 0.98 for glucose, 0.93 for ascorbic acid and 0.96 for uric acid. Notably, there was minimal interference from glucose, ascorbic acid and uric acid in the determination of H_2_O_2_. The good selectivity of this electrode is largely attributed to the low working potential (−0.25 V).

## 3. Experimental Section

### 3.1. Reagents and Chemicals

Double-stranded DNA (dsDNA, from Calf Thymus DNA; mol wt 8.0–15 kb, 41.9% GC ), HRP (E.C. 1.11.1.7, ≥250 U·mg^−1^, from horseradish), *d*-(+)-glucose, ascorbic acid and uric acid were all obtained from Sigma-Aldrich (Shanghai) Trading Co., Ltd (Shanghai, China). Chitosan was purchased from Sinopharm Chemcial Reagent Co., (Shanghai, China). Fe_3_O_4_ magnetic nanoparticles (~20 nm, 99.9%) was purchased from Beijing Dk Nano technology Co., Ltd. (Beijing, China). All chemicals were of analytical grade and used without further purification. The supporting electrolyte phosphate buffer was prepared by mixing the stock solutions of KH_2_PO_4_ and K_2_HPO_4_. Stock solution of 1 mg·mL^−1^ HRP was prepared by directly dissolving HRP in pH 7.0 phosphate buffer and stored at 4 °C. The Fe_3_O_4_ magnetic nanoparticles were dispersed in water by ultra-sonication for about 30 min and stored at 4 °C for use. The supporting electrolyte solution used in electrochemical impedance spectroscopy (EIS) measurements was 0.1 M KCl containing 5 mM [Fe(CN)_6_]^3−/4−^ (1:1). Double-distilled water was used throughout the experiments.

### 3.2. Preparation of DNA/Chitosan/Fe_3_O_4_/HRP/GCE

A GCE [3 mm diameter, Bioanalytical Systems (BAS), West Lafayette, IN, USA] was polished with 0.05 μm alumina slurry, rinsed with water, sonicated in water for 2 min, and dried. The DNA/chitosan/Fe_3_O_4_/HRP/GCE was prepared as follows: aqueous solutions of chitosan (0.5 mL, 1 mg/mL) and Fe_3_O_4_ (0.5 mL, 1 mg/mL) were mixed for 15 min. Then, 10 μL of dsDNA (1 mg/mL), 20 μL of the mixture of chitosan and Fe_3_O_4_, and 10 μL of HRP (1 mg/mL) aqueous solution were successively placed on the GCE surface to form a polyion complex layer. The electrode was allowed to dry for 24 h under a 1000 mL beaker at room temperature. After being rinsed with distilled water, the resulting DNA/chitosan/Fe_3_O_4_/HRP/GCE was stored in the refrigerator at 4 °C when not in use. Before electrochemical measurements, the electrodes were immersed in phosphate buffer for 15 min.

### 3.3. Electrochemical Measurements

All electrochemical experiments were performed with a conventional three-electrode system using a CHI 660D electrochemical workstation (Shanghai CH Instruments, Shanghai, China). An Ag/AgCl (sat. KCl) electrode, a platinum wire electrode (1 mm diameter) and a GCE were used as the reference electrode, the counter electrode, and the working electrode, respectively. CV and constant potential amperometry were carried out by using a deoxygenated (N_2_-saturated) 0.1 M phosphate buffer (10 mL). Deoxygenated electrolyte solutions were prepared by bubbling high purity grade nitrogen gas through the solution at least 20 min prior to the electrochemical measurements. EIS measurements were performed with 0.1 M KCl solution containing 5 mM [Fe(CN)_6_]^3−/4−^ under applied potential of 170 mV with the frequency range from 0.01 to 100,000 Hz with the amplitude of 8 mV. All measurements were done at room temperature.

### 3.4. Scanning Electron Microscopic Analysis

The SEM analysis of DNA/chitosan/Fe_3_O_4_/HRP polyion complex membrane was performed using a JEOL-6480LV microscope (JEOL Ltd., Tokyo, Japan) operating at 15.0 kV. Prior to SEM analysis, *ca*. 10 nm of carbon film was sputtered on the samples.

## 4. Conclusions

As one kind of novel nanomaterial, magnetic nanomaterials not only have the common nano-characters, but also have some special properties. Acting as the carrier of biological molecules, DNA/chitosan/Fe_3_O_4_ film can provide a microenvironment which is similar to biological molecules of internal environment. In this paper, a simple method for constructing HRP modified electrode on a DNA/chitosan/Fe_3_O_4_ bio-magnetic polyion complex membrane to realize direct electron transfer and electrocatalysis was proposed. The results show that HRP was successfully immobilized on the electrode surface by DNA/chitosan/Fe_3_O_4_ bio-polyion complex membrane. The HRP on the electrode exhibited fast electron transfer rate, high affinity to H_2_O_2_ and good bioactivity toward H_2_O_2_ reduction.
